# Impact of the COVID-19 pandemic on tuberculosis patients and tuberculosis control programs in Turkey, review and analysis

**DOI:** 10.1186/s13690-022-01007-w

**Published:** 2022-12-12

**Authors:** Sule Ozdemir, Derya Oztomurcuk, Muhammet Ali Oruc

**Affiliations:** 1 Department of Public Health, Faculty of Medicine, Samsun University, Samsun, Turkey; 2 Department of Public Health, Provincial Directorate of Health, Samsun, Turkey; 3 Department of Family Medicine, Faculty of Medicine, Samsun University, Samsun, Turkey

**Keywords:** Health policy, Tuberculosis control, COVID-19, Epidemiology, Pandemic

## Abstract

**Background:**

Many infectious diseases, including Tuberculosis (TB), have been put in the background with the COVID-19 pandemic. This study aimed to evaluate the changes in the number of TB patients, the parameters of the TB patients and tuberculosis control programs in the first year of the COVID-19 pandemic in Turkey when compared to the previous year.

**Methods:**

All TB patients who were recorded in Samsun province between March 1, 2019 and February 28, 2021 were included in this retrospective study. The data were analyzed in 2 groups as the COVID-19 period (March 2020 and February 2021) and the Pre-COVID-19 period (March 2019 and February 2020),the demographic and microbiological characteristics of the tuberculosis patients in both periods were compared according to months and years *p* < 0.05 was considered statistically significant.

**Results:**

The total number of TB patients was 320, although it was 172 in the Pre-COVID-19 period, it was 148 in the COVID-19 pandemic period. It was found that the TB incidence rate (IR) was 15.32%, the total number of examinations performed in TB dispensary decreased 33.54%, and the total number of contact examinations decreased by 53.54% during the pandemic period. The mean age of the patients decreased significantly during the COVID-19 period (*p* = 0.047), and it was found that culture positivity rates and smear positivity rates increased compared to the previous year (7.97%, *p* = 0.166, 1.86%; *p* = 0.507, respectively). SARS-CoV-2 PCR test result was found to be (−) in 46 (82.1%) of the 56 TB patients who were examined.

**Conclusions:**

In the present study, it was found that the incidence of TB, the number of examinations, and the number of contact examinations decreased at significant levels. The decrease in TB patients was mostly in the first 3 months when COVID-19 precautions and restrictions were intense. As a conclusion, it was observed that the application of TB patients to the healthcare institution and TB control were affected negatively by the COVID-19 pandemic.

## Background

According to the World Health Organization (WHO) 2020 global Tuberculosis (TB) report, approximately one-quarter of the world population was infected with TB bacillus. It is estimated that there were 10 million new TB patients worldwide in 2019 and 1.4 million people died from TB. TB maintains its importance as one of the top 10 mortality causes in the world [1]. In addition to this serious condition, many infectious diseases, including TB were pushed to the background because of the COVID-19 pandemic caused by SARS-CoV-2, which started in China and spread all over the world at the end of 2019. The WHO announced that there were an estimated 9.9 million people infected with TB in 2020 when the pandemic was still ongoing and 1.3 million deaths were because of TB [2].

It was predicted in a modeling study shared by the WHO that in case there is a global decrease of 25% in TB detection for 3 months with the COVID-19 pandemic, this will cause an increase of 13% in TB deaths, and a return to the TB death rates of 5 years ago would be possible globally [3]. It was also shared that an additional 1.4 million TB deaths may be recorded to existing deaths between 2020 and 2025 as a direct result of the pandemic [4]. According to the results of previous studies and the WHO, it was shown that the number of TB patients diagnosed in almost every country in 2020 decreased with the effects of the COVID-19 pandemic on the real-time fight against tuberculosis. As a result of the data collected by the Stop TB Partnership from 10 different global networks, significant decreases were seen in TB notifications and TB testing and diagnosis procedures in countries with a high TB ​​burden. It has been determined that TB patients have delays in applying to health institutions. The most important reason for this is that health institutions give priority to COVID-19 patients due to the pandemic. In addition, there have been delays in the stocking processes of TB drugs and due to the lack of sufficient health personnel in the COVID-19 response units, health personnel working in TB services had to work in these areas [1, 5]. As a result, the diagnosis, care and prevention of TB were impacted around the world. Most of the available data covers the first 6 months of the year 2020.There are relatively fewer data investigating the effects of the COVID-19 pandemic on TB patients for the second 6 months when it was expected that TB services would be restored and approximately 1 year after the restrictions began.

In the present study, the purpose was to evaluate the changes in the number of TB patients, the parameters of the TB patients and tuberculosis control programs in the first year of the COVID-19 pandemic in Turkey by comparing to the previous year and to define the variables of the TB patients with SARS-CoV-2 polymerase chain reaction (PCR) test results.

## Methods

The study was conducted in Samsun province, which is a city in the northern part of Turkey as a coastal city in the Central Black Sea region with a population of 1.356.079 in 2020 [6]. Tuberculosis control and other healthcare services provided in this city are similar to those available in other cities in Turkey [7].

The diagnosis of TB patients in Samsun province is made by the Training and Research Hospital (TRH) Chest Diseases Hospital of the university, private hospitals, state hospitals, and TB dispensaries in the city. The TRH Chest Diseases Hospital serves as a hospital with a special service for the hospitalization of TB patients at the city level. The patient follow-up of all diagnosed patients is provided by 3 TB dispensaries of the Ministry of Health at the provincial and district level. Also, there is a Coordination Unit in the city center that monitors the notification of TB patients residing in Samsun and receiving treatment as registered in the dispensary and receiving treatment under the Ministry’s guidelines [8].

In the study, the characteristics such as age, gender, nationality, case definition, place of involvement, diagnosis method, bacteriology and culture results, resistance profile and the total number of examinations and contacts registered in the dispensaries of TB patients who were analyzed retrospectively. The data were taken from the National TB System (UTS), a web-based program for TB patient records, and from the patients’ files in the dispensary. As well as the TB patient data, data such as the status of SARS-CoV-2 PCR testing, the result and date if performed, the presence of symptoms (at least one of the symptoms of COVID-19), the number of COVID-19 contacts per patient are also provided has been researched. This review was performed via the Public Health Management System (HSYS), which is a web-based software that collects national COVID-19 data. The first COVID-19 case in our country was announced on March 11, 2020. The TB patients registered to the dispensaries in the city and received treatment were examined in 2 groups as 1 year before the pandemic (Pre-COVID-19 period: 1 March 2019–29 February 2020) and 1 year after the onset of the pandemic (COVID-19 Period: 1 March 2020–28 February 2021). A second analysis was also performed for the 3-month periods of the 1 year as of the beginning of the pandemic (1st Period: March–April-May 2020, 2nd Period: June–July-August 2020, 3rd Period: September–October-November 2020, 4th Period: December 2020-January-February 2021). The annual incidence rate (IR) of TB was also calculated over the total population of the city on the specified dates. The incidence rate ratio (IRR) was used to compare the increase or decrease in the number of cases.

### Statistical analysis

The SPSS 21.0 package program was used for the statistical analysis and the results were expressed using mean ± standard deviation, median (interquartile range: IQR) and number (%) according to whether the data were parametric or not. The Chi-Square Test was used for categorical comparison of the groups and Student-t Test was used for the difference between two independent means for comparison in terms of characteristics determined by measurement. The statistical significance level for all tests was accepted as *p* < 0.05.

## Results

The total number of the TB patients who were included in the dates determined in the study was 320 (n:172 in the Pre-COVID-19 period, n:148 in the COVID-19 pandemic period). While the TB IR was 12.01 per 100,000 person-years in the Pre-COVID-19 period, the TB IR was found to be 10.17 per 100,000 person-years during the COVID-19 pandemic period. The IRR was 0.99 (95% CI (0.41–2.36). When the COVID-19 period was compared with the Pre-COVID-19 period, it was found that there was a 15.32% decrease in the incidence of TB, 33.54% in the total number of examinations performed in TB dispensaries, 53.54% in the total number of contact examinations and 46.55% in the number of contacts per patient (Table 1).

**Table 1:** Variables of patients registered in tuberculosis (TB) dispensaries, Samsun province

Variables	Pre-COVID-19 period	COVID-19 period	Percentage difference between periods (increase-decrease)
TB IR (per 100,000)^a^	12.01	10.17	−15.32%
Total number of examinations(n)	23,230	15,439	−33.54%
Total number of contact examinations(n)	2994	1391	−53.54%
The number of contacts per patient(n)	17.4	9.3	−46.55%

*IR* incidence rate, ^a^Samsun province population in 2019 (*n*) =1,348,542, Samsun province population in 2020 (*n*) = 1,356,079

**Fig. 1:**
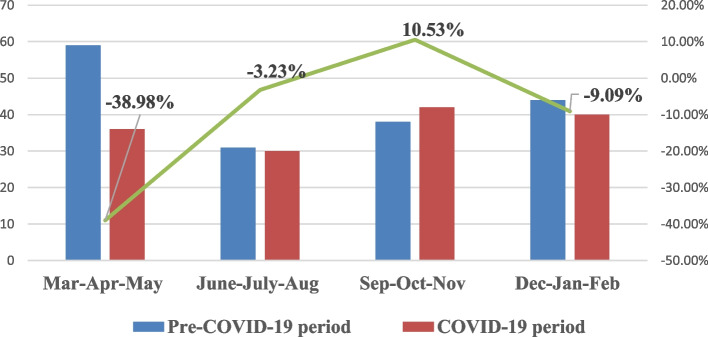
The distribution of the number of TB patients according to 3-month periods

The distribution of the number of patients according to 3-month periods is given in Fig. 1. Although the total number of registered TB patients in the first 3 months of the COVID-19 period (n: 36) was found to be less than the total number of registered TB patients (n: 59) in the first 3 months of the Pre-COVID-19 period, the number of registered TB patients in the other 3-month periods were found to be similar (*p* = 0.241).

The mean age of the patients in the COVID-19 period was found to be 48.74 ± 21.73 years, which was lower than the mean age of the patients in the Pre-COVID-19 period (53.44 ± 20.46 years) (*p* = 0.047). It was found that the frequency of pulmonary TB (PTB) patients during the COVID-19 period (71.6%) increased compared to the pre-COVID-19 period (69.8%), but there was no statistically significant difference (*p* = 0.717). It was determined that the frequency of multidrug-resistant TB (MDR-TB) patients (1.4%) decreased in the COVID-19 period compared to the pre-COVID-19 period (3.1%) (*p* = 0.448). Also, patients under 18 years of age with a diagnosis of TB (50.0%) and Republic of Turkey non-citizen (23.5%) patients were found to have increased in the period of COVID-19 (*p* = 0.196, *p* = 0.235, respectively) (Table 2).

**Table 2:** Demographic and microbiological characteristics of TB patients before and during COVID-19

Variables	Pre-COVID-19 period		COVID-19 period		Percentage difference between periods (increase-decrease)	p*
	*n* = 172		*n* = 148			
Age (years) Mean ± SD(median (IQR))	53.44 ± 20.46 (57.0(38.0–69.0))		48.74 ± 21.73 (53.0(30.0–65.0))			0.047**
	n	%^a^	n	%^a^		0.196
Age group (years)						
< 18	8	4.7	12	8.1	50.00%	
18–64	103	59.9	95	64.2	−7.77%	
> 65	61	35.5	41	27.7	−32.79%	
Gender						0.751
Male	105	61.0	88	59.5	−16.19%	
Female	67	39.0	60	40.5	−10.45%	
Country of origin						0.235
Turkey	155	90.1	127	85.8	−18.06%	
Others (Syria, Iraq, Iranian et al..)	17	9.9	21	14.2	23.53%	
TB site						0.717
Pulmonary	120	69.8	106	71.6	−11.67%	
Extra-pulmonary	52	30.2	42	28.4	−19.23%	
Patient type						0.773
New	162	94.2	138	93.2	−14.81%	
Relapse	8	4.7	9	6.1	12.50%	
Patient coming from treatment abandonment or failure	2	1.2	1	0.7	−50.00%	
Multi-drug resistance (MDR) results						0.448
No	94	96.9	69	98.6	−26.60%	
Yes	3	3.1	1	1.4	−66.67%	
TB diagnosis						0.070
Microscopy (smear) positive	49	29.1	46	30.4	−6.12%	
Culture positive	48	27.9	42	28.4	−12.50%	
Clinical or radiological positive	30	16.9	25	17.6	−16.67%	
Histopathological positive	45	26.2	29	19.6	−35.56%	

*The Chi-Square Test, **Student-T test, ^a^column percentage

It was found that the culture positivity rate among the cultured patients increased by 7.97% in the COVID-19 period compared to the Pre-COVID period (*p* = 0.166), and the microscopy (smear) positivity rate in the microscopy (smear) patients increased by 1.86% compared to the previous year (*p* = 0.507).

It was found in the analysis in which the COVID-19 pandemic period was evaluated that TB patients who were newly diagnosed with TB and had SARS-CoV-2 PCR test were mostly in the 3rd period (September–November) (*p* = 0.298, *p* = 0.615, respectively) (Table 3). As of March 2020, SARS-CoV-2 PCR test was detected as (−) in 46 (82.1%) of 56 TB patients who were tested for SARS-CoV-2 PCR test. The microscopy (smear) and culture positivity rates were found to be significantly decreased in the December–February period of the pandemic compared to previous periods (*p* = 0.003, *p* = 0.037, respectively) (Table 3).

**Table 3:** Demographic and microbiological characteristics of TB patients (n: 148) according to 3-month periods during the COVID-19 Pandemic period

Variables	Mar-Apr-May 2020 (1st Period)		June–July-Aug 2020 (2nd Period)		Sep-Oct-Nov2020 (3rd Period)		Dec 2020-Jan-Feb2021 (4th Period)		p*
	n:36	%^a^	n:30	%^a^	n:42	%^a^	n:40	%^a^	
Patient type									0.298
New	32	88.9	28	93.3	42	100.0	36	92.3	
Relaps	4	11.1	2	6.7	0	0.0	3	7.7	
TB site									0.847
Pulmonary	26	72.2	20	66.7	32	76.2	28	70.0	
Extra-pulmonary	10	27.8	10	33.3	10	23.8	12	30.0	
Microscopy (smear) results									0.003
Positive	13	50.0	14	73.7	11	40.7	5	19.2	
Negative	13	50.0	5	26.3	16	59.3	21	80.8	
Culture results									0.037
Positive	25	96.5	19	100.0	19	76.0	18	81.8	
Negative	1	3.5	0	0.0	6	24.0	4	18.2	
SARS-CoV-2 PCR test									0.615
Tested	10	27.8	12	40.0	18	42.9	16	40.0	
No tested	26	72.2	18	60.0	24	57.1	24	60.0	
SARS-CoV-2 PCR test									0.833
Positive	1	9.1	2	16.7	4	22.2	3	20.0	
Negative	10	90.9	10	83.3	14	77.8	12	80.0	

*The Chi-Square Test, ^a^column percentage

The distribution of tuberculosis patients (n:56) who underwent SARS-CoV-2 PCR test according to the variables was examined. It was found that SARS-CoV-2 PCR test was performed in 78.6% (n:44) of the patients before TB. Although 35 (62.5%) of them had symptoms, only 4 (7.1%) had a history of contact with a person with COVID-19 infection. When PTB patients were compared with EP-TB patients, no significant differences were detected in the (*p* = 0.200). However, the mean time between the first SARS-CoV-2 PCR test and the date of TB diagnosis of PTB patients (41.17 ± 42.58, median = 23.0 (7.0–61.0)) was compared to those with EP-TB (63.44 ± 57.41, median = 53.0 (13.0–88.0)) were found to be significantly shorter (*p* = 0.017). In patients who had TB diagnosis and SARS-CoV-2 PCR test (+) (n:10), the number of TB contacts per patient was 8.50 ± 10.76 (median = 5.5 (3.0–7.0)), the number of COVID-19 contacts was found to be 1.50 ± 1.17 (median = 1.0 (1.0–3.0)) people. The COVID-19 PCR test positive (n:10) status of TB patients whose TB diagnosis was confirmed histopathologically and bacteriologically was investigated. According to the time of TB diagnosis, 3 (30%) were found to have positive COVID-19 PCR test before TB diagnosis and 7 (70%) after TB diagnosis.

## Discussion

As far as we are concerned, this is the first study conducted in Turkey to evaluate TB case detection in the Pre-COVID-19 period and COVID-19 period, the parameters of patients diagnosed with TB and tuberculosis control programs. In this study, we found that there was a serious decrease in the incidence of TB during the COVID-19 pandemic period, an increase in the number of TB patients under the age of 18, and a decrease in the average age. Although there were decreases or increases in demographic and microbiological characteristics of TB patients, it was also observed that there were no significant differences in the majority of them.

The healthcare resources have been re-allocated all over the world, both human and economic resources were transferred to this area because of the importance and priority of the COVID-19 pandemic in terms of public health, and other healthcare services have been disrupted [9]. The WHO Global TB report for 2021 aims to decrease the TB incidence rate by 4–5% annually until 2020. However, it was reported that the decreased incidence of TB (18%) in 2019–2020 was very high with the COVID-19 pandemic [2]. In the TB report of the Republic of Turkey Ministry of Health, Turkey’s incidence of TB in 2017 was 14.6 (per hundred thousand), and our province was 13.5 (per hundred thousand). The incidence of TB in Turkey and our province in the last 5 years decreased by 3–5% on average every year [10]. In our study, it was found that the incidence of TB during the COVID-19 period decreased by more than 15% compared to the Pre-COVID-19 period, and the total number of examinations and the total number of contacts in the our province dispensaries decreased by approximately 30–50%. Similar to the WHO 2021 TB Global report [2], the rate of decrease in TB patients according to years is more pronounced during the pandemic period. There was a decrease in patient diagnosis especially in the first 6 months of the first year of the COVID-19 period in a study conducted in Malawi [11], in another study conducted by Mwamba et al. [12], they found that hospital visits of patients diagnosed with TB during the pandemic process decreased because of the fear of contracting COVID-19 and the high contagiousness of the disease. In China, in a study conducted by Wang et al. [13], it was determined that there was a decrease of more than 60% in the months of 2020, when the measures were intense, compared to the same period of the previous 3 years. The diagnosis, treatment and care services in our province during the pandemic process continued as before the pandemic in TB dispensaries and the TRH Chest Diseases Hospital in the city center. The striking decrease in the incidence of TB and the number of examinations may be because of the inability of the patients to be diagnosed, especially because they applied late to the health institution or did not apply. In our country and all over the world, we believe that the strict implementation of the measures and restrictions in the first months of the pandemic period (especially Mar-Apr-May) is effective in the decrease in the application of patients to the health institution. One of the critical components in TB control is accurate and effective contact screening by healthcare staff working in TB, which prevents the spread of the disease by early detection of active TB patients with secondary origin [14]. As a result of our study, it was found that the total number of contacts recorded in TB dispensaries and the number of contacts per patient decreased during the pandemic. With the pandemic, health personnel all over the world and in our country have been mobilized to be a part of the COVID-19 teams. The assignment of healthcare staff working in TB dispensaries in the city in various fields related to COVID-19 may have negatively affected this situation.

The WHO reports that the majority of TB cases (70%) occur between the ages of 15–54 [1]. The results of previous studies conducted in our country and all around the world also confirm that TB continues to be detected in young middle ages [1, 10]. In the present study, the mean age was similar to the literature data. It was found in the study of Aznar et al. conducted in Spain that the mean age of the patients was young to middle age, however, the mean age increased during the pandemic (median = 47.5) when compared to the pre-pandemic period [9]. Unlike the literature data, it was found in the present study that the mean age of TB cases in our city decreased at significant levels in the pandemic period. It is also possible to argue that this is because of the increase in TB cases under the age of 18 and the decrease in cases over the age of 65. In line with the precautionary decisions of the Ministry of Health and the Provincial Governorship, citizens under the age of 20 and over the age of 65 were prohibited from going out of their residence, walking around in open areas, parks, and traveling by public transport, and going out on the streets as of March 21, 2020 until May 31, 2020. And, as of June 1, 2020 to March 1, 2021, citizens who were over the age of 65 were allowed to go out at certain times of the day, although the rules were not as strict as in the first 3 months [15]. The measures had positive effects on the number of COVID-19 cases. However, many reasons such as the continuation of restrictions, transportation difficulties, fear of being infected with COVID-19 in healthcare institutions, stigma, ignoring the disease, and low clinical symptoms affected the admission to hospitals, decreasing the number of TB patients who were diagnosed over 65 years of age. Due to the increase in domestic contacts of possible TB patients, the number of pediatric TB patients diagnosed under the age of 18 may have increased. We specifically examined patients under the age of 18 during the COVID-19 period and the pre-COVID-19 period. As a result of screening contacts of patients with tuberculosis during the COVID-19 period, it was determined that the majority of patients under the age of 18 were diagnosed. This rate was higher than the previous year. We thought that the number of children under the age of 18 who were diagnosed with tuberculosis may have increased due to the increase in household contacts of potential tuberculosis patients. However, we did not have any data, except we knew that there was a contact between the retrospective data of the patients. Therefore, future qualitative and quantitative studies with TB patients will help assess how much TB healthcare delivery was affected and the reduction in TB cases during the pandemic.

The rate of foreign nationals among total TB patients has been increasing in recent years in our country [10]. The increased number of immigrants and refugees who come to our country for reasons such as economic or war conditions is also effective in this. In our study, compared to the Pre-COVID-19 period, it was found that the ratio of non-citizen patients among all TB patients increased. The number of foreign TB patients may have increased during the pandemic process as the lack of primary protection measures quarantine and isolation opportunities for immigrants and refugees left them vulnerable to COVID-19 and tuberculosis [16]. It must also be noted that necessary treatment and care services are provided free of charge to every patient identified as a TB case in our country including foreign nationals [8]. However, more problems may be experienced in the diagnosis, treatment, and care services of TB because of the socioeconomic variability of foreign national patients.

It was found that there was a two-fold decrease in EP-TB cases compared to PTB cases during the pandemic period. The diagnosis of EP-TB cases is more difficult than PTB and histopathological examination is required for the diagnosis [17]. The decrease in the rate of patients diagnosed histopathologically in our study may explain the decrease (19.6%) in EP-TB case rates during the pandemic period. In the 1st 3 months of the pandemic, to reduce the impact of the pandemic on health resources in our country, restrictions were imposed on elective and outpatient services, focusing on the continuation of emergency and basic clinical services, which are priority areas in the healthcare system [18]. In this process, the limited use of standard surgical procedures may have been effective in reducing the histopathological diagnosis and for this reason, the patients with EP-TB diagnosis. It was found in two separate studies conducted in Zimbabwe [19] and China [13] that the rate of bacteriological (smear) positivity was significantly higher when the pandemic period was compared with the pre-pandemic period. In our study, which is similar to the literature, it was found that the rate of culture and microscopy (smear) positivity increased during the pandemic period. The increase in the bacteriological positivity rate may be because of the increase in hospital admissions of cases with serious symptoms and requiring medical attention [13].

According to the data of the Ministry of Health, The COVID-19 cases in our country increased significantly after the first 6 months of the pandemic [20]. In our study, we found that the most (32.7%) TB patients were tested for the SARS-CoV-2 PCR test after the 6th month of the pandemic. Although approximately 62% of TB patients who underwent SARS-CoV-2 PCR test had symptoms, very few of them had positive (17.9%) SARS-CoV-2 PCR tests. Similarities in symptoms of tuberculosis and COVID-19 may require simultaneous diagnostic testing for both TB and COVID-19. Talodini et al. [21] found in their study with 49 patients who had simultaneous TB and COVID-19 infection that 53% had TB before COVID-19 and 28% had initial COVID-19 infection. However, because of the low number of cases here, future studies on the coexistence of TB-COVID-19 infection in the coming years will help us better know the long-term epidemiological impact of COVID-19 measures.

Low-income countries and middle-income countries face worsening health system due to poor living conditions, lack of sanitation, limited access to health facilities and population growth. In addition, the COVID-19 epidemic and the gradual quarantine implemented in countries adversely affected tuberculosis and individuals receiving routine TB services. For this reason, we would like to focus on the possible long-term impact on TB control in our country. Due to the needs that emerged with the pandemic, the health workforce and resources gave priority to COVID-19 disease instead of routine services within the health system. These practices had a negative impact on TB patients and their households, resulting in a decrease in TB testing rates and delays in diagnosis and treatment. Of course, fewer TB diagnoses and less compliance in TB treatment will result in worsening treatment outcomes, increased transmission rates, and increased TB burden and TB-related mortality in the long run. In order to help mitigate the impact of the pandemic on TB control, there is a need for planning that strengthens health systems for COVID-19 and other possible causes of pandemics in the future.

The strength of our work was that it was a retrospective analysis of real-time surveillance data of pandemic and pre-pandemic TB patients in a province of Turkey. The data were cross-checked by the TB monitoring and evaluation officer of the province (TB Provincial Coordinator), and the data in the web-based system (UTS) of the country’s TB registries. The limitation of our study was that the data of TB patients were collected in a city in Turkey and therefore, the obtained results may not represent Turkey. Also, another important limitation was that the success rates of TB treatment during the COVID-19 pandemic were not yet known. During the pandemic period, TB healthcare workers in our city continued to distribute anti-tuberculosis drugs uninterruptedly and the majority of patients were controlled with video-surveillance treatment (e-DOT). Studies that show the results of patients who continue TB treatment in the future are needed to find the success rates of TB treatment during the pandemic period.

## Conclusions

As a result of the study, it was found that there was a decrease in the incidence of TB, the total number of examinations performed in TB dispensaries and the total number of contacts in the first year of the COVID-19 pandemic, compared to the previous year. Although wearing face masks and taking social distance measures because of COVID-19 contributed to reducing tuberculosis transmission in the community, the decrease in the number of TB patients was greater, especially in the first 3 months of the pandemic, when COVID-19 restrictions were intense. These results show that with the COVID-19 pandemic, the application of TB patients to the health institution and the TB control were negatively affected. Multi-faceted measures should be taken to reduce the negative effects and the conditions provided in the pre-pandemic period should be returned to TB services.

## Data Availability

Not applicable.
